# Distal Femoral Replacement Versus Open Reduction and Internal Fixation in Elderly Distal Femur Fractures: A Systematic Review and Meta-Analysis

**DOI:** 10.7759/cureus.99012

**Published:** 2025-12-11

**Authors:** Ahmed Elnewishy, Mohamed Elgamal, Ahmed Elkohail, Mahmoud Noureldin, Ahmed Hamada, Pir Zarak Khan, Hagar Teama, Symeon Naoum

**Affiliations:** 1 Trauma and Orthopaedics, Royal Berkshire NHS Foundation Trust, Reading, GBR; 2 Trauma and Orthopaedics, Southend University Hospital, Southend-on-Sea, GBR; 3 Trauma and Orthopaedics, Princess Royal University Hospital, King's College Hospital NHS Foundation Trust, Orpington, GBR; 4 Trauma and Orthopaedics, University Hospitals Sussex NHS Foundation Trust, Brighton, GBR; 5 Trauma and Orthopedic, Royal Devon and Exeter Hospital, Exeter, GBR; 6 Trauma and Orthopaedics, Maidstone and Tunbridge Wells NHS Trust, Royal Tunbridge Wells, GBR; 7 Pharmacy, Kafr El-Sheikh University Hospital, Kafr El-Shaikh, EGY; 8 Trauma and Orthopaedic Surgery, Royal Berkshire NHS Foundation Trust, Reading, GBR

**Keywords:** distal femoral replacement (dfr), distal femur fracture, elderly trauma, intraarticular distal femur fracture, morbidity and mortality, open reduction internal fixation

## Abstract

Distal femur fractures are a major cause of morbidity and mortality among older adults, often complicated by osteoporosis and comorbidities that hinder recovery. This systematic review and meta-analysis compared distal femoral replacement (DFR) and open reduction internal fixation (ORIF) for such fractures in elderly patients. A comprehensive search of PubMed, Scopus, Google Scholar, and the Cochrane Library identified six comparative studies including 688 patients. Pooled data showed a trend toward fewer reoperations with DFR (odds ratio (OR): 0.44, 95% confidence interval (CI): 0.16-1.21) and similar complication rates between DFR and ORIF (OR: 0.96, 95% CI: 0.37-2.54). Mortality was significantly lower in patients treated with DFR (OR: 0.32, 95% CI: 0.12-0.84). These findings suggest that DFR may reduce mortality and the need for revision surgery without increasing complications, supporting its consideration as an alternative option for elderly patients with distal femur fractures.

## Introduction and background

Distal femur fractures (DFFs) represent an important clinical problem in older adults, largely due to age-related osteoporosis and the expanding elderly population. These injuries typically follow low-energy falls from standing height and occur more often in elderly women because of reduced bone mineral density [1]. Their incidence rises sharply after age 60, with a marked female predominance [2]. Mortality remains high, with reported annual rates between 18% and 35%, comparable to hip fractures [3]. Recent Medicare data also highlight a substantial increase in DFF incidence among individuals aged 85 years and older between 2006 and 2019 [4].

Management of these fractures is challenging because osteoporotic bone increases the risk of fixation failure, malunion, and nonunion [5], while comorbidities such as cardiovascular disease, dementia, and frailty further complicate perioperative care and rehabilitation [3]. Despite advances in fixation techniques, postoperative problems including stiffness, immobility, and infection remain frequent [6]. Achieving stable fixation is particularly difficult in comminuted or periprosthetic fractures, prompting the use of constructs such as dual plating or nail-plate combinations [7]. Even with successful fixation, elderly patients often have delayed functional recovery, reduced quality of life, and persistently high mortality, reported between 10% and 38% annually [8].

Open reduction and internal fixation (ORIF) has traditionally been the standard treatment for DFFs, using locking plates or intramedullary devices to achieve anatomical reduction and promote healing [9]. However, outcomes in elderly patients vary. One study reported good-to-excellent Western Ontario and McMaster Universities Osteoarthritis Index (WOMAC) scores in 91% of cases despite a 32% reoperation rate due to issues such as nonunion and implant prominence, while another large investigation showed high 30-day morbidity and mortality influenced by age, comorbidities, and pre-injury functional status [10].

Distal femoral replacement (DFR) has emerged as an alternative option for complex DFFs in elderly patients, aiming to provide immediate stability and facilitate unrestricted early mobilisation [11]. Reports show rapid postoperative recovery, with early ambulation within days after surgery and favourable knee range of motion in selected frail individuals. DFR has been described as particularly suitable for highly comminuted or osteoporotic fractures in medically complex patients where early mobilisation may reduce immobility-related risks [12].

Comparative research evaluating DFR versus ORIF in elderly patients has produced mixed findings. A systematic review of 1,258 fractures found no significant difference in reoperation or surgical complication rates, although DFR demonstrated a higher rate of medical complications such as infection [13]. Another meta-analysis reported similar reoperation, complication, and functional outcomes between both treatments, noting only slightly better knee motion with ORIF and emphasising the need for further high-quality comparative studies [14].

Review objective

This study seeks, through systematic review and meta-analysis, to evaluate and compare the clinical effectiveness of DFR and open reduction with internal fixation (ORIF) for DFFs in geriatric populations.

Materials and methods

Search Strategy

In September 2025, a comprehensive literature search was done using PubMed, Scopus, Google Scholar, and the Cochrane Library to locate investigations comparing DFR and ORIF in geriatric DFFs. The search incorporated MeSH terms and keywords including “distal femur fracture”, “distal femoral replacement”, “distal femoral arthroplasty”, “open reduction internal fixation”, “locking plate”, and “retrograde intramedullary nail”. Boolean logic (AND, OR) was employed to refine queries, with limits applied to English-language human studies. Manual screening of references from included papers and related systematic reviews was conducted to ensure completeness. This systematic review followed the Preferred Reporting Items for Systematic Reviews and Meta-Analyses (PRISMA) 2020 guidelines [15].

Inclusion Criteria

Eligible studies were retrospective or prospective comparative investigations that directly evaluated DFR against ORIF in patients aged ≥65 years presenting with osteoporotic, comminuted, or periprosthetic DFFs. Eligible studies had to report at least one primary clinical endpoint such as reoperation, complication rate, or mortality, using clearly defined outcome criteria. Only full-text English-language articles were considered.

Exclusion Criteria

Studies without direct comparison between DFR and ORIF were excluded. Case reports, small case series, conference abstracts, narrative reviews, editorials, and commentaries were not eligible. Publications lacking extractable outcome data or not available in full text were also excluded.

Outcome measures

Primary endpoints included rates of reoperation, overall complications (infection, thromboembolic events, malunion, nonunion, implant failure), and all-cause mortality at defined follow-up intervals. Secondary endpoints included functional outcomes (Oxford Knee Score (OKS), Knee Society Score (KSS), Parker Mobility Index (PMI), and range of motion), resource utilization (length of stay, transfusion requirement, cost), and perioperative variables. Functional outcomes reported in the included studies were assessed using validated instruments: the OKS [16], KSS [17], and PMI [18]. These instruments were not administered by our team; we extracted published scores from the included studies. Secondary synthesis of published instrument scores does not require additional licensing for non-commercial academic use.

Data extraction and quality assessment

Two reviewers independently carried out data extraction using predesigned standardized forms. The collected information covered study design, sample size, patient demographics, fracture classification, surgical method, follow-up duration, and reported clinical outcomes. Conflicts were resolved through mutual agreement or, if necessary, by a third reviewer. Methodological quality was assessed using the Newcastle-Ottawa Scale (NOS) [19], which evaluates selection, comparability, and outcome domains.

Statistical analysis

All statistical procedures were conducted using Review Manager software (RevMan, version 5.4; The Cochrane Collaboration, London, UK) [20]. Dichotomous variables - including reoperation, complication, and mortality rates - were synthesized as odds ratios (ORs) with 95% confidence intervals (CIs). Both fixed-effect and random-effects models were employed to ensure robustness of pooled estimates. Between-study heterogeneity was quantified using the I² statistic, with values >50% interpreted as substantial heterogeneity. Visual examination of funnel plots was performed to detect potential publication bias and confirmed with Egger’s regression test, applying a threshold of p < 0.05 for significance.

## Review

Search and study selection

**Figure 1:**
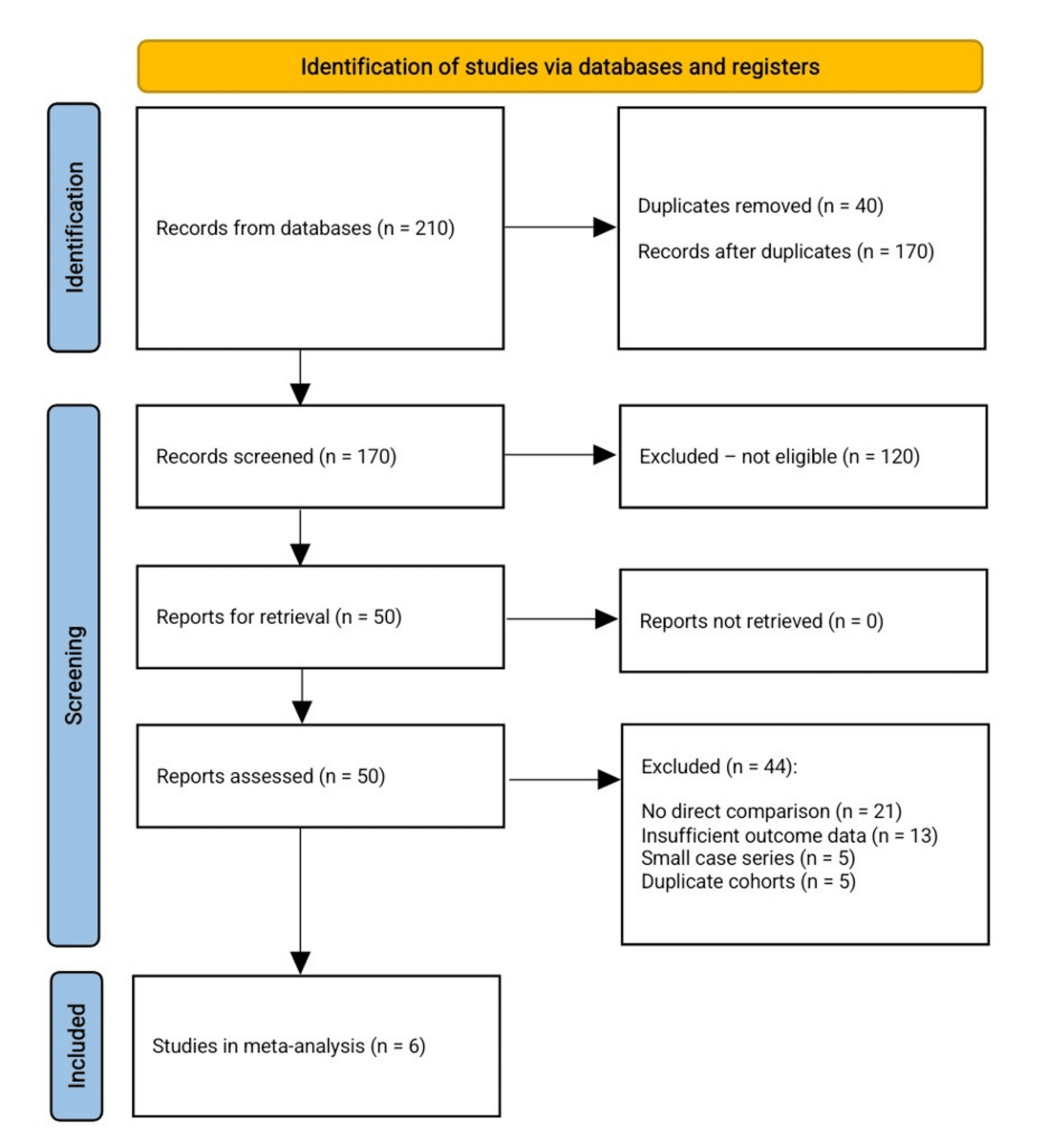
PRISMA flow chart for the included studies PRISMA: Preferred Reporting Items for Systematic Reviews and Meta-Analyses [15]

The structured literature search identified 210 articles. After the removal of 40 duplicates, 170 unique records were screened. Title and abstract review excluded 120 studies that did not meet eligibility (direct comparison of DFR versus ORIF in elderly or periprosthetic distal femur fractures). Fifty full texts were assessed; 44 were excluded for no direct DFR-ORIF comparison (n=21), inadequate outcome reporting (n=13), case series with <10 patients (n=5), or duplicate cohorts (n=5). Six studies met all criteria and were included in the meta-analysis (Figure 1).

Study characteristics

Six retrospective comparative studies evaluated DFR versus ORIF in elderly distal femur fractures (total n=688; study sizes: 27-363). Cohorts were predominantly older adults (mean age: 74-85 years) with female predominance (≈70-90%). Both native and periprosthetic fractures after total knee arthroplasty were represented. Interventions were DFR (including distal femoral arthroplasty) versus ORIF (locking plates or retrograde intramedullary nails). Perioperative care was standardised within studies, but surgical techniques varied. Follow-up ranged from 30 days (database studies) to ≥12 months (institutional cohorts). Outcomes included mortality (30-day/1-year), reoperation, complications (nonunion, infection, malunion, DVT/PE), selected functional scores-OKS [16], KSS [17], PMI [18], and resource measures (length of stay, transfusion, cost) (Table 1).

**Table 1:** Key characteristics of the included studies comparing distal femoral replacement (DFR) versus open reduction and internal fixation (ORIF) in elderly distal femur fractures (DFFs) Abbreviations: DFR, distal femoral replacement; ORIF, open reduction internal fixation; LOS, length of stay; ROM, range of motion; KSS, Knee Society Score; OKS, Oxford Knee Score; PMI, Parker Mobility Index; TKA, total knee arthroplasty Sources: Oxford Knee Score (OKS) [16] , Knee Society Score (KSS) [17] , and Parker Mobility Index (PMI) [18]

Category	Study Design	Sample Size (DFR/ORIF)	Level of Evidence	Patient Demographics	Intervention Details	Follow-up Duration	Outcome Measures	Results	Complications	Conclusion
Ang et al. [21]	Retrospective observational study (single institution, 5-year)	14/13	Level IV	Mean age: 85 (DFR) vs 80 (IF); 89% female; mean interval from primary TKA to fracture: 80 months (0–181)	DFR: distal femoral replacement. IF: internal fixation (methods not detailed)	5 years	LOS, ITU stay, re-fixation, mortality	LOS: 56 vs 55 days. ITU: 3 patients (DFR). Re-fixation: 1 (IF). 30-day mortality: 1 (DFR). 1-year mortality: 7% (DFR) vs 15% (IF)	No intraoperative complications; higher ITU requirement in DFR	Similar LOS. IF group had higher 1-year mortality and one re-fixation. DFR linked to greater ITU use
Garabano et al. [22]	Retrospective cohort study (two centers)	20/55	Level III	Median age: 79 (ORIF) vs 85 (DFR); 80% women; high ASA (III–IV); osteoporosis common	ORIF: plates, screws, nails, dual constructs. DFR: cemented hinged prosthesis with femoral/tibial stems; immediate full WB	Mean 32 months (ORIF) vs 40 months (DFR)	PMI, ROM, union, reoperations, mortality	DFR better PMI at 1–3 months; no difference at 12 months. Union: 93% ORIF at 4.9 months. Mortality: 4% (30d), 20% (1y)	ORIF 38% complications vs 10% DFR (p=0.022). ORIF: nonunion, fixation failure, infection. DFR: 2 periprosthetic fractures	DFR → faster short-term recovery, fewer complications. Long-term outcomes, reoperation, mortality similar
Gwam et al. [23]	Retrospective national database (ACS-NSQIP, NIS; matched 1:2)	121/242	Level III	Mean age 69.6 ± 14; 83.7% female; matched for BMI, ASA, comorbidities	ORIF: CPT 27511, 27513, 27514. DFR: CPT 27442, 27443, 27445, 27447. Excluded periprosthetic	30 days	Mortality, readmission, reoperation, complications, transfusion, discharge, cost	DFR → higher transfusion (57% vs 44.2%, p=0.021) & cost ($46k vs $20k, p<0.001). No difference in reoperation, readmission, discharge. Subgroup ≥80 yrs: lower 30d mortality (0% vs 18.2%) and readmission (0% vs 18.2%)	Higher transfusion & costs with DFR. In octogenarians, DFR reduced mortality/readmission vs ORIF	DFR increases costs but improves survival in ≥80 yrs
Hart et al. [24]	Retrospective cohort study (single institution)	10/28	Level III	Mean age ≈ 82; majority female; low-energy falls main cause	ORIF: locking plates. DFR: modular distal femoral prostheses	1 year (union FU up to 128 weeks)	Reoperation, union, ambulation, living status, mortality	Nonunion 18% ORIF. Reoperation: 11% ORIF vs 10% DFR. At 1 year, all DFR ambulatory vs 23% ORIF wheelchair dependent	ORIF: 1 superficial, 1 deep infection. DFR: 1 DVT, 1 superficial, 1 deep infection	DFR avoided nonunion, preserved ambulation. No mortality/reoperation difference
Tandon et al. [25]	Retrospective cohort (UK)	21/40	Level IV	DFR: mean 78 yrs, 18F/3M. ORIF: mean 74 yrs, 31F/9M. Low-energy trauma common	DFR: arthroplasty via anterior midline approach. ORIF: 23 plates, 17 retrograde nails	≥72 months	LOS, WB time, OKS, KSS, VAS, ROM	LOS: 9 days (DFR) vs 32 (ORIF). WB: 1.5 days vs 11 weeks. Functional scores similar. Costs ~£9600 vs £9800	DFR: 2 deaths, 1 superficial infection, 1 DVT. ORIF: 6 deaths, 6 malunions, 1 nonunion, 2 infections	DFR enabled earlier mobilization, shorter LOS, fewer complications; functional scores/costs similar
Tibbo et al. [26]	Retrospective matched cohort	10/20	Level IV	Mean age: 80 (DFR) vs 76 (ORIF); ~60% female; BMI ~30	DFR: cemented rotating hinge prostheses. ORIF: locking plates, LISS, NCB plates, nails	Mean 20 months (DFR) vs 29 (ORIF)	Survivorship, reoperation, LOS, EBL, transfusion, ROM, KSS	2-yr revision-free survivorship: 90% DFR vs 65% ORIF. Reoperation-free: 90% vs 50%. ORIF: 15% nonunion, 10% delayed union. Functional outcomes similar. DFR had higher blood loss (592 vs 364 mL, p=0.02) and longer LOS (13 vs 6.5 days, p=0.04)	ORIF: 3 nonunion, 2 delayed union, 2 malunion, 1 infection. DFR: 1 revision (patellofemoral impingement)	Both had similar function. DFR ↓ reoperations but ↑ blood loss and LOS

Quality assessment of the included studies

Study quality was evaluated using the NOS [19], which evaluates study quality across key domains, such as cohort selection, comparability, and outcome assessment. Each study was categorized as having low, moderate, or high quality according to its scores in these domains. Table 2 presents the individual study scores.

**Table 2:** Quality assessment of the included studies using the Newcastle-Ottawa Scale (NOS) ★ indicates a low score for the respective category. ★★ represents a moderate score, reflecting acceptable quality. ★★★ denotes a high score, indicating strong quality in the respective domain. Source: Newcastle-Ottawa Scale [19]

Study (Author, Year)	Selection	Comparability	Outcome	Total score (out of 9)
Ang et al. [21]	★★	★	★	4
Garabano et al. [22]	★★★★	★★	★★★	9
Gwam et al. [23]	★★★★	★★	★★	8
Hart et al. [24]	★★★	★	★★	6
Tandon et al. [25]	★★★	★	★★	6
Tibbo et al. [26]	★★★	★★	★★	7

Results of the meta-analysis

Comparison of DFR and ORIF for Reoperation Rates

Forest plot analysis examined the risk of reoperation following DFR compared with ORIF in elderly distal femur fractures. The pooled odds ratio (OR) was 0.44 (95% CI: 0.16-1.21), suggesting a trend toward lower reoperation risk with DFR, though the difference did not reach statistical significance (p=0.11).

**Figure 2:**
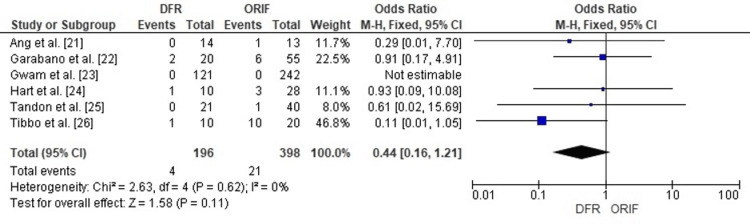
Forest plot comparing the reoperation rates in patients treated with DFR versus ORIF for distal femur fractures DFR: Distal Femoral Replacement; ORIF: Open Reduction and Internal Fixation; OR: Odds Ratio; CI: Confidence Interval Source: [21-26]

No heterogeneity across the included studies (I²=0%), demonstrating uniform results across cohorts despite variation in fracture type (native vs periprosthetic) and study design (single-center vs database). These results imply that while DFR may reduce the likelihood of reoperation compared to ORIF, the evidence remains limited by small sample sizes in individual studies (Figure 2).

Publication Bias Assessment for Reoperation Rates

**Figure 3:**
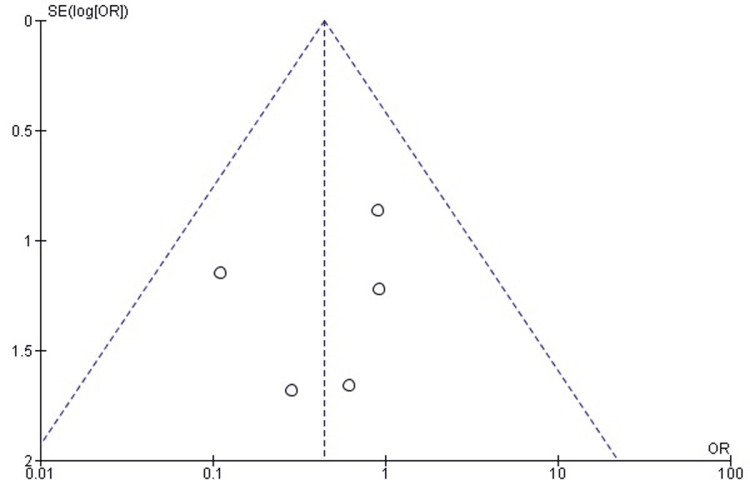
Funnel plot assessing publication bias for reoperation outcomes in studies comparing DFR and ORIF for distal femur fractures DFR: Distal Femoral Replacement; ORIF: Open Reduction and Internal Fixation; OR: Odds Ratio; CI: Confidence Interval; SE: Standard Error Source: [21-26]

Funnel plot evaluation demonstrated that studies were evenly distributed around the pooled estimate, with no signs of small-study effects or bias in publication. Egger’s test was non-significant (p > 0.05), confirming that the observed distribution was unlikely to be due to selective reporting (Figure 3).

Comparison of DFR and ORIF for Complication Rates

Forest plot analysis compared complication rates between DFR and ORIF in elderly distal femur fractures. The pooled OR was 0.96 (95% CI: 0.37-2.54), revealing no meaningful statistical distinction between DFR and ORIF (p=0.94).

**Figure 4:**
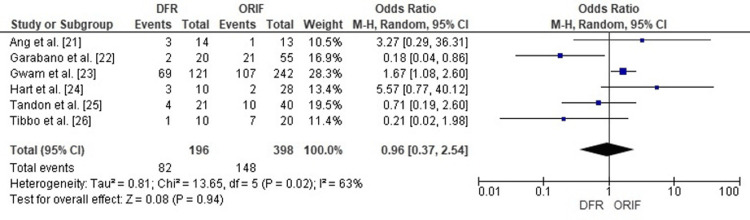
Forest plot comparing complication rates in patients treated with DFR versus ORIF for distal femur fractures DFR: Distal Femoral Replacement; ORIF: Open Reduction and Internal Fixation; OR: Odds Ratio; CI: Confidence Interval Source: [ 21-26 ]

Moderate heterogeneity was observed (I²=63%), reflecting variability in complication definitions across studies (infection, thromboembolic events, malunion, nonunion, implant failure). Despite this, the overall pooled analysis suggests that both DFR and ORIF carry comparable risks of complications in elderly distal femur fractures (Figure 4).

Publication Bias Assessment for Complication Rates

**Figure 5:**
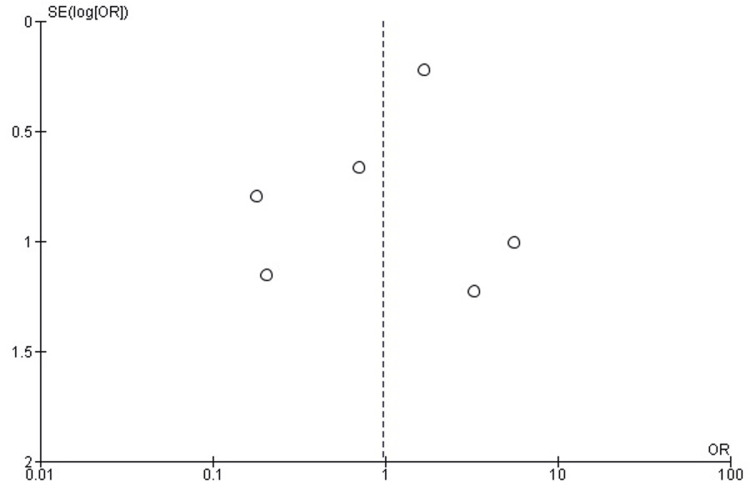
Funnel plot assessing publication bias for complication outcomes in studies comparing DFR and ORIF for distal femur fractures DFR: Distal Femoral Replacement; ORIF: Open Reduction and Internal Fixation; OR: Odds Ratio; CI: Confidence Interval; SE: Standard Error Source: [21-26]

Funnel plot analysis demonstrated an approximately symmetrical distribution of studies, with no strong evidence of publication bias. Egger’s regression test was non-significant (p>0.05), indicating that the asymmetry may be attributable to inter-study differences rather than selective reporting (Figure 5).

Comparison of DFR and ORIF for Mortality

Forest plot analysis assessed mortality rates following DFR compared with ORIF in elderly distal femur fractures. The pooled OR was 0.32 (95% CI: 0.12-0.84), demonstrating a statistically significant reduction in mortality for patients treated with DFR (p=0.02).

**Figure 6:**
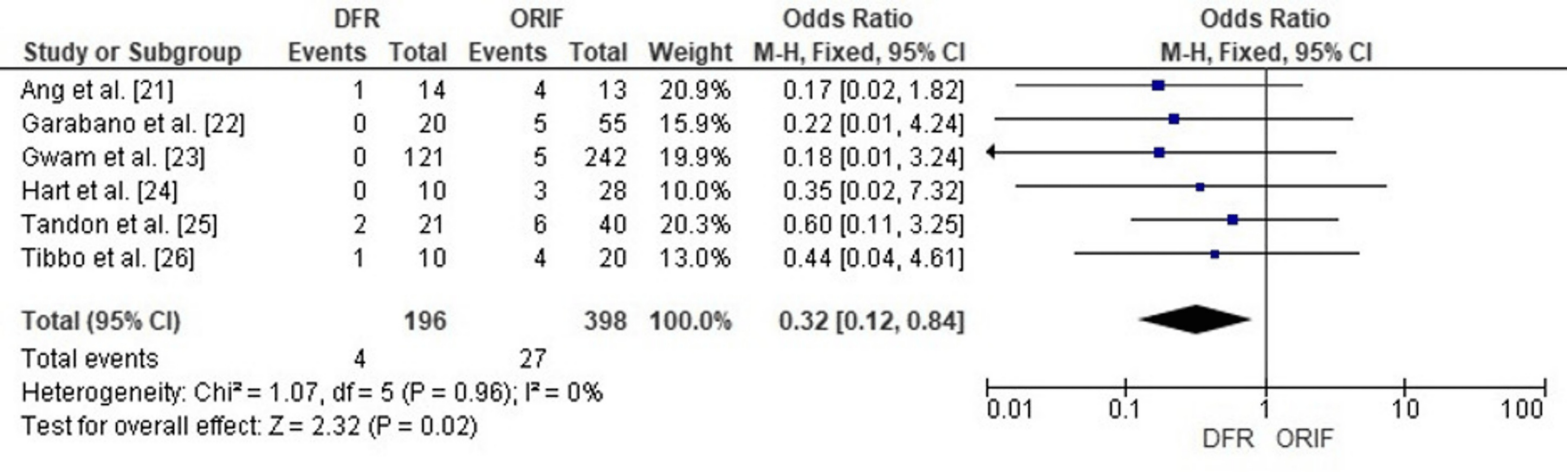
Forest plot comparing mortality rates in patients treated with DFR versus ORIF for distal femur fractures DFR: Distal Femoral Replacement; ORIF: Open Reduction and Internal Fixation; OR: Odds Ratio; CI: Confidence Interval Source: [21-26]

No heterogeneity among studies (I²=0%), indicating consistency across single-center cohorts and database analyses. These findings suggest that DFR is associated with a lower risk of mortality compared to ORIF in the elderly population with distal femur fractures (Figure 6).

Publication Bias Assessment for Mortality

**Figure 7:**
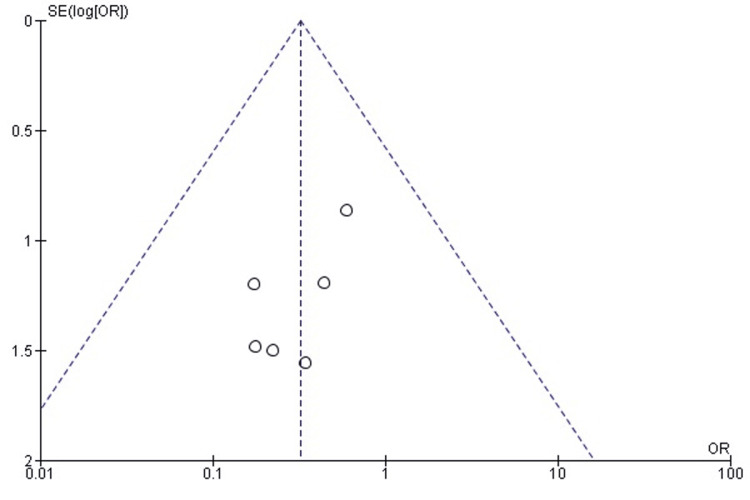
Funnel plot assessing publication bias for mortality outcomes in studies comparing DFR and ORIF for distal femur fractures DFR: Distal Femoral Replacement; ORIF: Open Reduction and Internal Fixation; OR: Odds Ratio; CI: Confidence Interval; SE: Standard Error Source: [21-26]

Funnel plot analysis demonstrated symmetrical distribution of studies around the pooled effect estimate, with no evidence of publication bias. Egger’s regression test confirmed non-significant findings (p>0.05), indicating that observed patterns are unlikely to result from selective reporting (Figure 7).

Discussion

Comparison of DFR and ORIF remains important in elderly distal femur fractures. ORIF has traditionally been the preferred approach, while DFR offers immediate mechanical stability and usually allows early mobilisation. This meta-analysis summarises available evidence from retrospective comparative studies.

Pooled findings showed a nonsignificant trend toward fewer reoperations with DFR, suggesting a potential reduction in fixation-related failures, although this signal should be interpreted cautiously due to limited data. Tibbo et al. reported higher two-year revision-free survivorship with DFR (90%) compared with ORIF (65%), with fewer nonunions and delayed unions. Hart et al. [24] similarly observed an 18% nonunion rate after ORIF but none after DFR. In contrast, Gwam et al. [27] did not identify early differences in reoperation using national data, indicating that any divergence may become more apparent at longer follow-up. Overall, although DFR may lower the need for later revision, conclusions remain restricted by heterogeneity and retrospective designs.

Complication rates were generally comparable between groups. Garabano et al. [22] observed fewer complications with DFR (10% vs 38%), largely due to avoidance of fixation failure and nonunion. Tandon et al. [25] also noted earlier weight bearing (1.5 days vs 11 weeks) and fewer malunions and infections in the DFR cohort. Conversely, Tibbo et al. [26] and Ang et al. [21] found DFR associated with greater blood loss, longer hospitalisation, and higher intensive care utilisation, and Gwam et al. [27] reported increased transfusion requirements and overall cost. These findings indicate that, while DFR reduces fixation-related complications, it also introduces perioperative burdens that must be weighed carefully [28].

Mortality outcomes consistently favoured DFR across multiple studies. Ang et al. [21] reported one-year mortality of 7% with DFR versus 15% with ORIF. Gwam et al. [27] found lower 30-day mortality and readmission among octogenarians treated with DFR, and Tandon et al. [25] similarly reported fewer long-term deaths in the DFR cohort. Although causal mechanisms cannot be confirmed due to retrospective methodology, these observations suggest that the ability to mobilise earlier with DFR may reduce complications associated with prolonged immobility, such as pneumonia and thromboembolism [29].

Functional recovery also appeared to favour DFR in the short term. Garabano et al. [22] demonstrated higher early PMI scores that equalised by 12 months. Tandon et al. [25] reported shorter length of stay (9 vs 32 days) and earlier mobilisation, and Hart et al. [24] showed all DFR patients were ambulatory at one year, compared with 23% wheelchair dependence in the ORIF group. However, Gwam et al. [27] noted that these functional gains come with increased operative time, transfusion rates, and cost. Thus, DFR offers earlier functional improvement at the expense of greater perioperative resource utilisation [30].

Overall, the findings are consistent with previous meta-analyses showing similar complication and reoperation rates between DFR and ORIF but emphasising DFR’s potential to facilitate earlier mobilisation and reduce mortality. This review supports those trends, although the evidence remains limited, and reoperation findings did not reach statistical significance [31].

Limitations

All included studies were retrospective, which introduces selection bias and confounding. Significant heterogeneity existed across studies in fracture patterns (native and periprosthetic), surgical techniques, implant types, and definitions of complications. Follow-up varied widely, from 30 days to over one year, and economic outcomes were inconsistently assessed. Reoperation rates after ORIF may be underestimated, as frail patients with malunion or nonunion are often not considered suitable for revision surgery, which may obscure true differences between treatments. The small number of eligible comparative studies further limits certainty and emphasises the need for well-designed prospective research to clarify the long-term comparative effectiveness of DFR and ORIF in elderly distal femur fractures.

## Conclusions

This meta-analysis found that complication rates were similar between DFR and ORIF, with a non-significant trend toward fewer reoperations and a consistent association with lower mortality in patients treated with DFR. While these findings may reflect the benefits of immediate stability and earlier mobilisation, they should be interpreted conservatively given the retrospective study designs, variability in fracture patterns and surgical techniques, and the small number of eligible comparative studies. Reoperation rates after ORIF may also be underestimated in frail patients who are not considered suitable for revision. High-quality prospective research is needed to clarify long-term outcomes, better define the indications for DFR, and determine which patients are most likely to benefit from each treatment approach.
